# Mapping the Knowledge of Research Trends in Sports Performance Asymmetries from 2015 to 2024: A Bibliometric Study and Analysis of the Most-Cited Papers

**DOI:** 10.3390/sports13040093

**Published:** 2025-03-24

**Authors:** Boryi A. Becerra-Patiño, Juan David Paucar-Uribe, Jorge Olivares-Arancibia, Alex Ojeda-Aravena, Rodrigo Yáñez-Sepúlveda, José M. Gamonales, José Francisco López-Gil, Víctor Hernández-Beltrán

**Affiliations:** 1 Faculty of Physical Education, National Pedagogical University, Bogotá 110221, Colombia; babecerrap@pedagogica.edu.co (B.A.B.-P.); jdpaucaru@upn.edu.co (J.D.P.-U.); 2 AFySE Group, Research in Physical Activity and School Health, School of Physical Education, Faculty of Education, Universidad de las Américas, Santiago 7500975, Chile; jolivares@udla.cl; 3 Departamento de Ciencias de la Actividad Física, Universidad de Los Lagos, Osorno 1305, Chile; alex.ojeda@ulagos.cl; 4 Faculty Education and Social Sciences, Universidad Andres Bello, Viña del Mar 2520000, Chile; rodrigo.yanez.s@unab.cl; 5 Training Optimization and Sports Performance Research Group (GOERD), Faculty of Sport Science, University of Extremadura, 10005 Cáceres, Spain; martingamonales@unex.es (J.M.G.); victorhb@unex.es (V.H.-B.); 6 Faculty of Education and Psychology, University of Extremadura, 06071 Badajoz, Spain; 7 One Health Research Group, Universidad de las Américas, Quito 170124, Ecuador

**Keywords:** dominance, interlimb strength, muscular speed, asymmetry, performance, limb differences

## Abstract

In recent years, studies related to preventing injuries and improving sports performance have aroused academic interest. However, no bibliometric study has investigated asymmetry. The aim of this study was twofold: (i) to identify trends in research on asymmetries in sports performance through bibliometric analysis, and (ii) to determine the most-cited articles to establish the main lines of research on asymmetries. The final sample consisted of 471 documents. The results show that, during the publication period, there was a considerable increase (73%) in research between 2020 and 2021, with 2022–2023 being the year with the highest production and number of citations. Most of the publications consisted of research articles (93.41%), with a low percentage of review studies (4.24%). The Journal of Strength and Conditioning Research and Symmetry were the journals with the highest number of documents (*n* = 57) and citations (*n* = 1230). The journals that produced the most knowledge were those in the first quartile (Q1) and the second quartile (Q2). The most prolific author was Bishop, C. The countries with the highest number of published documents were England (140 documents and 3039 citations) and the United States (94 documents and 2099 citations). The most common words in the studies were performance (*n* = 162), strength (*n* = 167), reliability (*n* = 118), injury (*n* = 94), and asymmetry (*n* = 90). The study of asymmetries in sports performance has focused on two main approaches: one related to analyzing differences between limbs, and the other focused on assessing strength after postoperative processes. The analysis of the existing body of knowledge on asymmetries allows us to incorporate the latest scientific advancements. In turn, this helps us to establish best practices to enhance both sports performance and rehabilitation processes.

## 1. Introduction

According to Maloney [1], symmetry is defined as the quality of equivalence between two identical halves that meet exact sizes and shapes. Science has demonstrated an interest in analyzing its impact on human movement for more than three decades [2]. The multiple athletic gestures of athletes are established based on the specificity of the sport, which is a fundamental factor for determining patterns of repeated or changing movements [3]. Furthermore, the physical demands on athletes are conditioned by technical and tactical actions [4], which has generated growing interest within the sports science community to understand the effects and influence of physical variables on performance [5]. As a result, research on lower limb asymmetries has gained significant importance in recent years as an object of study in various combat sports [6]; cooperative–oppositional sports such as basketball [7], football [8,9], and volleyball [10]; and roller sports such as skating [11] and hockey [12]. Similarly, the effect of asymmetry has been studied in various sports that display a high number of accelerations (ACCs), decelerations (DECs), and changes in direction (CODs), such as tennis, football, and basketball [13].

Asymmetries are considered variables that directly influence athletes’ sports performance, as a variability of 10% [14] or 15% [15] could affect the risk of injury. Therefore, to reduce the likelihood of injury, the analysis of symmetries is crucial to quantify athletes’ performance. Initially, the use of asymmetries has allowed the correlation of strength and power training through jump tests to determine sports injuries [16] and the decline in physical capacity [17]. However, there are inconsistencies in the published studies. For instance, one study revealed no significant effect on proximal groin injuries in professional football players (*p* = 0.09) [18], and despite the unclear evidence, continuing to investigate this phenomenon to consider how to provide more efficient rehabilitation for athletes is essential [19].

However, athletes who return to their sport after an anterior cruciate ligament (ACL) injury often exhibit significant asymmetries in vertical jump performance [20], which can be attributed to reduced neuromuscular response capacity and an impaired ability to effectively handle fall stimuli [21]. These asymmetries not only compromise athletic performance, but also increase the risk of re-injury or the development of compensatory movement patterns that may lead to secondary injuries. The use of orthopedic devices after an ACL injury has been found to be ineffective in preventing lower limb imbalances [22], generating interest in improving recovery processes for athletes following an injury [23].

In individual sports, a greater contribution from the ankle joint is required to increase efficiency and allow for a greater acceleration ability [24], modifying step length and athlete posture [25]. Moreover, physical capacities such as CODs are more noticeable in long-duration periods [26], and there are deficits in vertical jump height in the functional performance of the joint [27]. Conversely, in high-intensity sports involving repeated sprints, no anomalies have been observed [28]. Plyometric jump training (PJT) has shown positive correlations with athletes’ fitness levels [29]; however, to assess PJT, the differences between each lower limb should be considered [30]. A study with female soccer players concluded that CMJ height asymmetry evidenced no association with speed or CODS; however, DJ asymmetries were significantly associated with slower performances in the 10 m (r = 0.52; *p* < 0.05) and 30 m sprint tests (r = 0.58; *p* < 0.05) and 505 (r = 0.52–0.66; *p* < 0.05) [31]. A review conducted by Fox et al. [32] revealed that asymmetries between limbs negatively affect COD and sprint performance, but not vertical jump performance.

In cyclic sports in which running economy (RE) is critical for the outcome, torque imbalances do not allow for proper performance development [33]. Similarly, movements involving CODs, high-intensity speed, and jumps are influenced by differences greater than 5% [34]. Sports practices that require the use of both legs, but where preferences exist for specific exercises, an isokinetic torque evaluation is also required [35], with the latter being a fundamental tool for functional instability [36]; when applied to a limb with lower ability, it is more likely to increase its functionality [1]. Therefore, strength training should be based on the principles of individuality and specificity of sports demands [37]. The selection of the test should include factors such as sports conditions, instrument reliability, and association with injury risk [38,39,40]. Consequently, inappropriate implementation of the evaluation may lead to low reliability and high bias in the result, yielding values that are far from the analyzed data and are not truly representative [41].

Another study conducted by Barrera-Dominguez et al. [42] aimed to identify and analyze the mobility, dynamic balance, and torque of the lower limbs and the prevalence of asymmetry in basketball and handball athletes. The study concluded that asymmetries showed considerable dispersion that was not dependent on the type of sport, but rather on everyone’s response and the test applied. Therefore, in sports involving dynamic balance, the Star Excursion Balance Test (SEBT) [43] or Y-Balance Test (YBT) [44] is recommended, where asymmetries with differences >4 cm are predictors of injury [45].

Over time, various studies have evaluated asymmetries with strength training [46] and interventions related to strength performance [47], where tests such as bilateral drop jumps (DJs), unilateral and bilateral countermovement jumps (CMJs) [48], and squat jumps (SJs) [49] are the most prominent for determining performance status and identifying associations and correlations between capacities [50]. In conclusion, a systematic review determined the effects on physical and sports performance and concluded that increased imbalances have harmful effects, but there are difficulties in understanding these effects, which is why more research is needed [51]. Thus, the present study conducts a retrospective analysis of the scientific evidence on asymmetries in sports performance, highlighting countries, authors, citations, journals, and others, to establish a mapping of research trends between 2015 and 2024. To our knowledge, no bibliometric study has analyzed the scientific production of asymmetries in sports performance or analyzed the most-cited documents. Therefore, the aim of this study was twofold: (i) to identify trends in research on asymmetries in sports performance through bibliometric analysis (from 2015 to June 2024), and (ii) to determine the most-cited articles to establish the main lines of research on asymmetries.

## 2. Materials and Methods

### 2.1. Design

This study is based on a bibliometric analysis of the scientific production of asymmetries over the last 10 years (2015–2024), and uses bibliometrics as a research technique. In accordance with the classification of Passas [52], this study is considered theoretical research, where the main objective was to analyze and synthesize a large amount of information related to the number of publications made over time and their evolution to the present day [53]. This type of analysis through bibliometrics allows for the identification of important scientific production criteria, including institutions, researchers, countries, keywords, and citations [54,55], through qualitative analysis.

### 2.2. Data Extraction

The document search was conducted in mid-June, 2024, by the two main researchers (J.D.P.-U. and B.A.B.-P.), in the following databases: Web of Science (WoS), as it is the most commonly used database for bibliometric studies [56,57], and Scopus, owing to its multidisciplinary orientation [58] for studies on physiological, biomechanical, and biomedical variables, as well as nutrition and injuries. The review was carried out based on the PRISMA recommendations [59] via the following search phrase: (“Interlimb asymmetries” OR asymmetries OR strength) AND (“Sport Performance”). Additionally, the [Title/Abstract] filter was used to identify documents that included the terms in the title or abstract. Finally, 1042 documents were found on the WoS platform, and 1440 were found on Scopus. These results were analyzed and reviewed by the two main researchers of the study (J.D.P.-U. and B.A.B.-P.).

**Figure 1:**
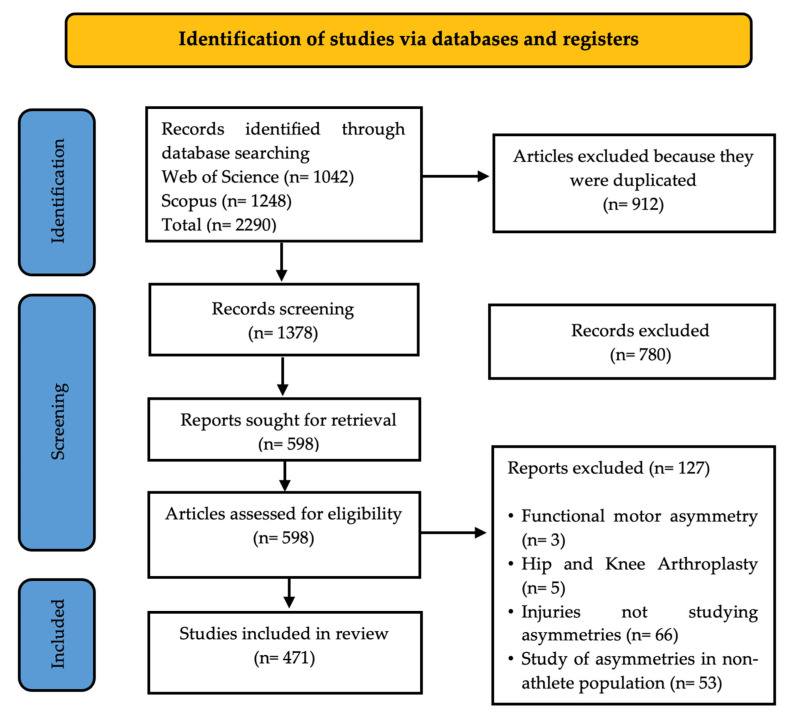
Flow diagram for selection of studies according to PRISMA guidelines.

A systematic process was developed through different stages for document selection based on bibliometric criteria [58]. A total of 2290 documents were identified. Afterwards, 598 documents were collected and subjected to metadata regulation to eliminate duplicates or documents that did not meet the inclusion criteria. Following the PRISMA procedure, only 471 documents remained that met the eligibility criteria established for this study (Figure 1). These documents were downloaded from WoS and Scopus in plain text and Excel formats for further analysis.

### 2.3. Eligibility Criteria

To select the most important and relevant studies related to the topic selected, several inclusion criteria were established: (i) studies of asymmetries in athletes in the respective categories (training, specialization, and/or elite); (ii) original full-text peer-reviewed studies; (iii) studies with access to the full document to complement the initial review; (iv) documents without language restriction; (v) studies with a publication date between 1 January 2015 and 15 June 2024; and (vi) studies from journals indexed in the Journal Citation Report (JCR). The exclusion criteria were as follows: (i) studies evaluating functional motor asymmetries; (ii) studies evaluating asymmetries in high school and/or university students who practice sports; (iii) injury studies not considering asymmetries; and (iv) studies evaluating imbalances but not considering asymmetries. These criteria respond to the need to be able to investigate the effects of asymmetries in sports performance.

### 2.4. Data Analysis

Data were extracted from the WoS and Scopus databases in two different formats: plain text and Excel. This action allowed for descriptive and percentage-based analyses of the results via a Microsoft Excel spreadsheet (v. 2006, Microsoft Corporation, Redmond, WA, USA). To classify the documents, an analysis matrix was created based on the following categories: (1) document type; (2) year of publication; (3) author names; (4) number of authors per study; (5) area of knowledge according to JCR; (6) article title; (7) journal name; (8) country of journal; (9) number of publications per year; (10) quartile based on Journal Citation Indicator (JCI) ranking; (11) total number of citations; (12) average number of citations per published article; (13) documents by institutional affiliation; (14) number of documents by department affiliation; (15) documents by publisher; and (16) number of documents by area of knowledge. On the other hand, the data downloaded in plain text format were analyzed with VOSviewer software (v.6.19, Center for Science and Technology Studies, The Netherlands), which creates two-dimensional graphs [60]. The primary purpose of this tool is to determine the exponential increase in scientific production in specific areas, considering the availability of information found in the consulted databases [61]. The graphs generated with VOSviewer considered the following categories: (1) occurrence by keywords; (2) citation by documents; (3) citation by journals; (4) citation by countries; (5) citation by authors; (6) cocitation by cited references; (7) cocitation by the minimum number of citations of a cited reference; (8) cocitation by the number of citations of a cited journal; and (9) cocitation by the number of citations of a cited author. A fragmentation analysis was performed with an attraction value of 3 and a repulsion value of −3. Additionally, the following basic laws of bibliometric analysis were used [62]: Price’s law, through the use of the R^2^ coefficient [63]; Lotka’s law, to identify the authors who had developed the most studies [64]; and Zipf’s law, to determine the terms with the highest occurrence [65]. The academic and research productivity of the authors was determined on the basis of the authors’ H-index, which establishes the number of h documents that have been cited a minimum of h times [66], as well as using Lotka’s law [67].

## 3. Results

The results are presented below based on bibliometric indicators.

### 3.1. Evolution in the Number of Publications

**Figure 2:**
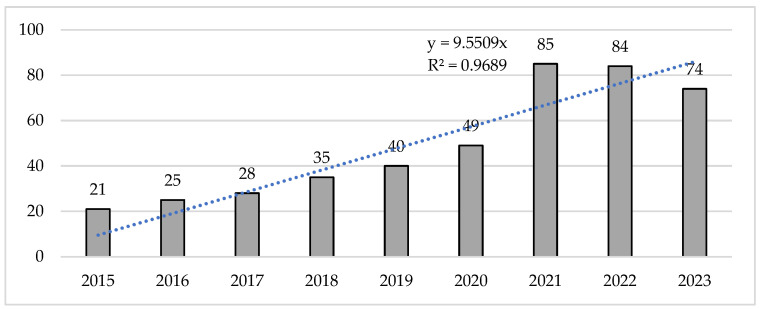
Evolution in number of documents per year.

The analysis of scientific production reveals how the number of publications per year has increased. There was exponential growth from the first year analyzed to the last year, with 2021 and 2022 being the years with the highest production (*n* = 169). With respect to the R^2^ coefficient of the sample, exponential growth in the sample was identified, with an increase of 304.76% in the number of publications, considering the number of papers developed from 2015 to 2021 (Figure 2).

### 3.2. Document Type

Considering the study design selected by the authors to carry out their investigations, the largest number of papers were developed as scientific articles. On the other hand, it is important to note the low number of review studies (Table 1).

### 3.3. Citation Analysis

**Figure 3:**
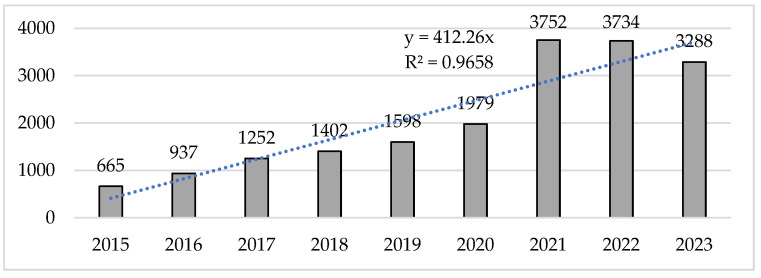
Number of citations per year.

On the other hand, the number of citations per year showed an exponential increase, especially between 2021 and 2022, which were also the years with the highest number of citations received (Figure 3). With respect to the total number of citations received by the studies in each of the years, a significant increase was observed, with 2021 and 2022 receiving totals of 3752 and 3734 citations, respectively. This increase in the number of citations allowed us to perform a collective analysis of the sample, which evidenced an exponential increase of 464.21% when comparing the citations received in 2015 and 2021, which was the year of the highest number of citations.

**Figure 4:**
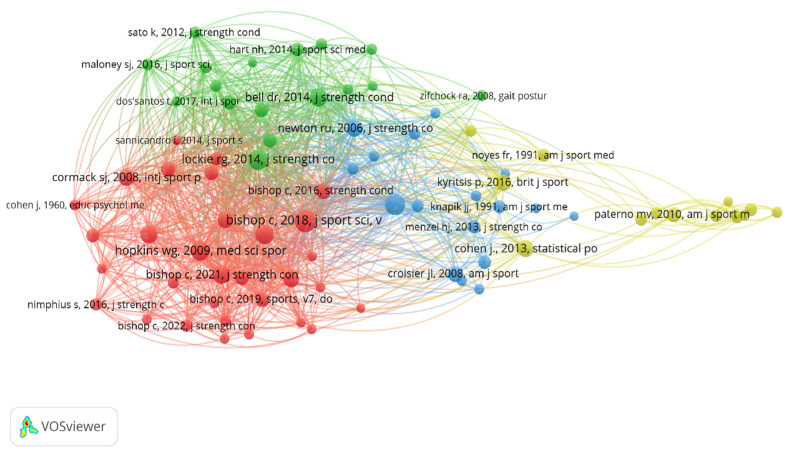
Analysis of the collaborative networks and interactions existing in the minimum number of citations of a reference that was cited for the study of asymmetries in sports performance.

For the analysis of citations from a cited reference, the minimum number of citations should be 20. Of the 10,252 references identified, only 79 exceeded this threshold. Notably, several studies had more than 100 citations [1,68,69,70,71,72,73,74,75]. Figure 4 shows the interactions produced between the references that were cited; the colors reflect these interactions.

After the documents were analyzed and classified, we determined the most-cited articles, which are presented in Table 2 and Table 3, arranged in descending order based on the total number of citations. Notably, two empirical research articles surpassed 200 citations each [69,70]. In comparison, there was only one literature review that exceeded this threshold, receiving a total of 248 citations since its publication [51].

**Figure 5:**
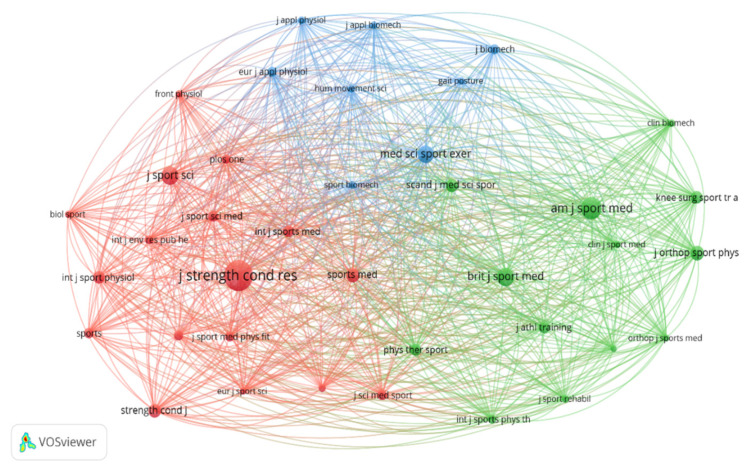
Collaborative network analysis of cocitations between leading journals for study of asymmetries in sports performance.

For the analysis of cocitations of the identified journals, a minimum of 100 citations was established for inclusion in the analysis. Of the 2052 journals identified, only 38 met this threshold. The journals *Journal of Strength and Conditioning Research* (2380 citations), *The American Journal of Sports Medicine* (1205 citations), *British Journal of Sports Medicine* (822 citations), and *Journal of Sport Sciences* (809 citations) stand out. Figure 5 shows three major nodes, with different colors highlighting the cocitation interactions between journals.

### 3.4. Authors’ Analysis

For the analysis of the documents by author, considering the first author, it is evident that the main authors were “Bishop, C.” and “Malý, T.”, who published the greatest number of studies. The top seven authors identified in this analysis each had a minimum of five published studies and contributed to 13.16% of the total scientific production (Table 4).

With respect to academic cooperation, which was evaluated with respect to the number of authors, it is evident that half of the total scientific production was carried out by teams of three to five authors. Interestingly, production by six to eight authors was greater than that by one to two authors, highlighting the importance of collaboration among researchers to generate new knowledge related to the study of asymmetries in sports performance (Table 5).

**Figure 6:**
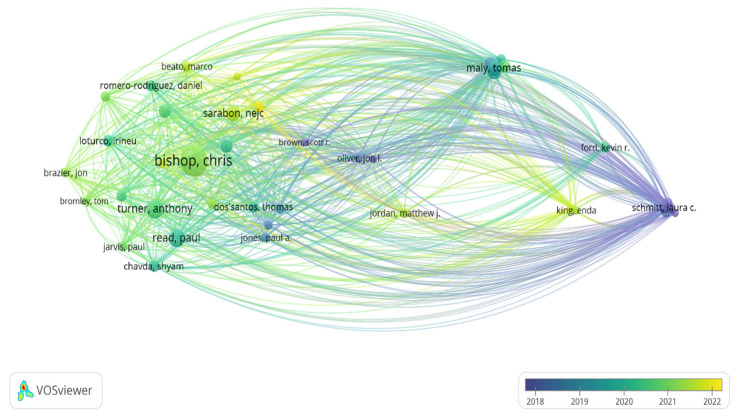
Analysis of collaborative networks and existing nodes among authors’ citations in study of asymmetries in sports performance.

Figure 6 shows the interactions between the authors. Three strong nodes can be observed: the node led by “Schitt, L.C.” from 2018, “Maly, T.” from 2019, and “Read, P.” and “Turner, A.”, who led from 2020 to 2021. On the other hand, the strongest node that can be identified is the one led by “Bishop, C.”, which was strengthened between 2021 and 2022. The size of the nodes represents the number of published documents, the lines correspond to the established connections, and the color indicates the temporal distribution of the publications. This image shows the authors who have been cited the most in reference to time, where an increased size of the node refers to a greater number of citations received.

**Figure 7:**
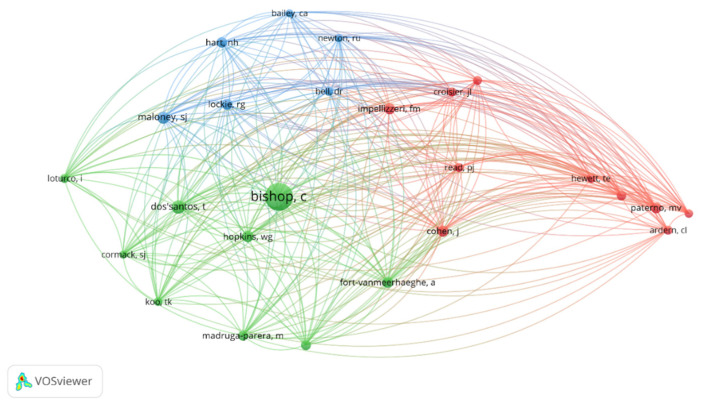
Analysis of collaborative networks and nodes for minimum number of citations in which an author has been cited.

The citation analysis of a cited author determined that of the 6679 identified authors, only 25 met the criterion of a minimum citation threshold of 60. Figure 7 highlights three strong nodes, with the green node being the strongest, led by “Bishop, C.”; the red node led by authors such as “Impellizeri, F.”, “Read, P.”, and “Paterno, M.”; and the blue node, the weakest of the three identified, consisting of “Maloney, S.”, “Lockie, R.”, and “Hart, N.”. Figure 7 reveals how there are three major nodes for the citation of a cited author, highlighting the green node centralized by Bishop, which, in turn, establishes connections with all the other authors.

### 3.5. Keyword Analysis

**Figure 8:**
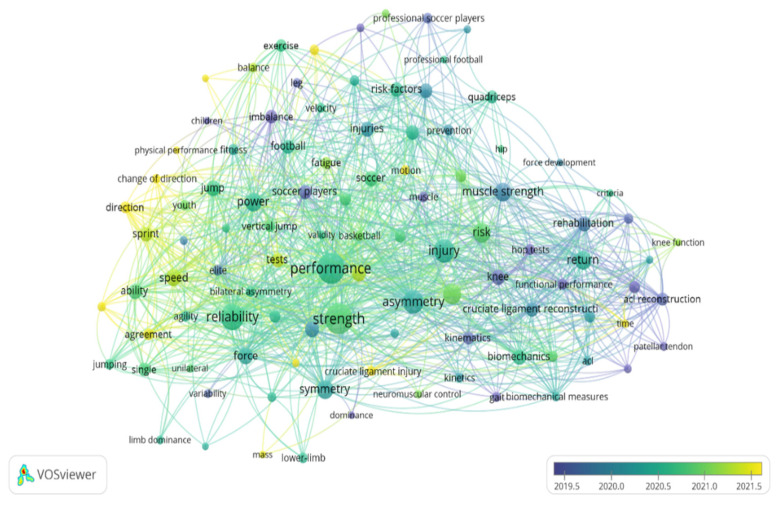
Collaborative network analysis of research keywords involved in asymmetries in sports performance.

For the keyword analysis, each term was required to have a minimum occurrence of 10. Of the 1635 collected keywords, only 102 met this criterion. The most frequently occurring keywords in the studies were “Performance” (*n* = 162), “Strength” (*n* = 167), “Reliability” (*n* = 118), “Injury” (*n* = 94), and “Asymmetry” (*n* = 90). Over time, the oldest concepts included “Imbalance”, “Soccer players”, “Muscle”, “Knee”, and “ACL reconstruction”. Moreover, between 2020 and 2021, the most referenced terms were “Performance”, “Injury”, “Return”, “Asymmetry”, “Symmetry”, and “Reliability”. The most recent terms used to study asymmetries in sports performance include “change of direction”, “sprint”, “speed”, and “physical performance” (Figure 8).

### 3.6. Analysis of the Journals

To establish the relationships between the names of the journals publishing scientific knowledge, the country, the number of publications, the category ranking, the JCI category for 2023, the quartile, the JCI index, the total number of citations, and the average number of citations, Table 6 was created. This table compiles the information corresponding to 212 documents out of the 471 eligible documents (45.01%). The top 12 journals producing scientific knowledge on the study of asymmetries in sports performance are from only four countries: the United States (25.05%), Switzerland (14.43%), the United Kingdom (3.60%), and Poland (1.91%). The journals with the highest number of published documents are from Europe and North America. There are no journals from South America, Asia, Africa, or Oceania. The journals publishing this knowledge are categorized into the Q1 and Q2 quartiles. Notably, Q2 journals publish the greatest number of documents, whereas Q1 journals receive the most citations, totaling 3434 for Q1, compared with 1191 for Q2. Among the nine journals with the highest number of published documents, most are related to the “Sport Sciences” category, except for three journals that cover other thematic areas also addressing the study of asymmetries. Notably, the two journals with the highest production are the “Journal of Strength and Conditioning Research”, with 57 documents and an average of 21.57 citations per article, and the “Symmetry Basel”, with 27 documents and an average of 4.37 citations per article. Other journals with high average citation levels per article include the “American Journal of Sports Medicine” (*n* = 72.58), “Journal of Sport Sciences” (*n* = 37.96), and “Strength and Conditioning Journal” (*n* = 33.80).

**Figure 9:**
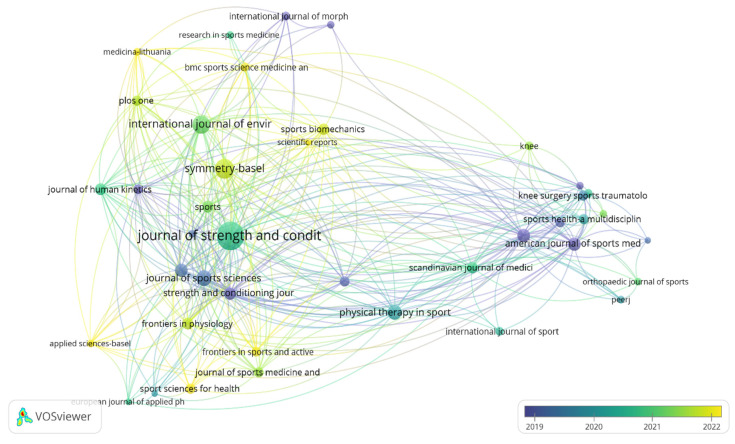
Visualization of maps and clusters of most-cited journals related to study of asymmetries in sports performance.

A total of 117 journals published 1691 documents related to the analysis of asymmetries in sports performance. For the citation analysis by journal, a journal was considered if it had published at least three documents and received 10 citations; thus, only 41 journals met this threshold. The main journal connections over time have been established, with the *Journal of Sport Sciences* and *Journal of Strength and Conditioning Research* being the most prominent in 2019, and the *Journal of Strength and Conditioning Research* leading between 2020 and 2021. Additionally, several journals have been at the forefront of producing knowledge on the study of asymmetries in sports performance since 2022, including *Symmetry-Basel*, *Sports Biomechanics*, *Scientific Reports*, *BMC Sports Science and Medicine*, *Medicina-Lithuania*, *Applied Sciences-Basel*, and *Frontiers in Sports and Active Living*. The different-sized nodes represent the journals, and the colors represent the interactions produced between them over time, with the darkest colors indicating citations established in 2019, and green indicating the most recent citations (Figure 9).

### 3.7. Editorial Analysis

The scientific production carried out by the publishers disseminating scientific knowledge reveals that “Lippincott Williams & Wilkins” and “MDPI” are the publishers with the greatest number of documents published, accounting for 35.03% of the total scientific production. Specifically, there are two publishers with more than 1000 citations: “Lippincott Williams & Wilkins” (*n* = 1974 citations) and “Sage Publication LTD” (*n* = 1007 citations). Notably, the top 10 publishers contributed to 70.91% of the total documents analyzed. Finally, five publishers have low citation levels: “Human Kinetics Publ Inc”, “Elsevier”, “Frontiers Media SA”, “Wiley”, and “Edizion Minerva Medica”, with fewer than 320 citations (Table 7).

### 3.8. Knowledge Areas

The analysis of the knowledge areas producing research on asymmetries and sports performance, based on the published documents, revealed that the main area was “Sport Sciences”. Similarly, the top two areas, “Sport Sciences” and “Orthopedics”, accounted for more than half of the published documents (56.89%) (Table 8).

### 3.9. Language and Country Analysis

In terms of language, English was the predominant language used, followed by Portuguese. Moreover, only two documents were found in languages other than those mentioned. Of the 471 documents, only six were published in other languages (three in Spanish, two in German, and one in Portuguese). The citation analysis by country revealed that only 59 countries have contributed to the knowledge of asymmetries. The criteria established were that each country should have published at least three studies and received 10 citations for inclusion in the analysis; only 40 countries met this threshold.

**Figure 10:**
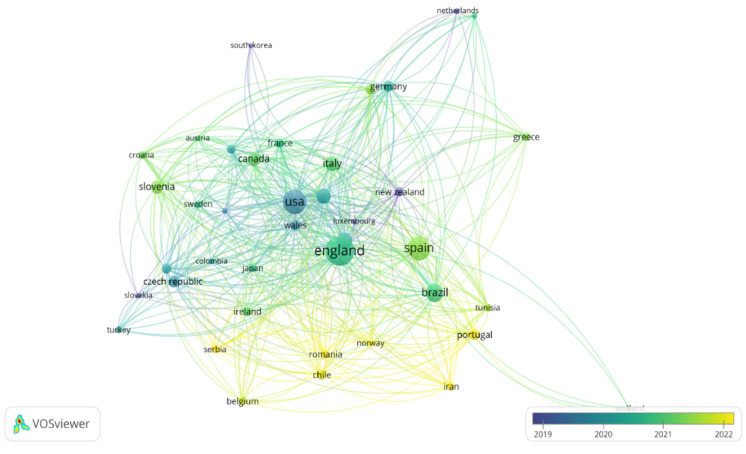
Visualization of maps and clusters of most productive countries/regions related to study of asymmetries in sports performance.

The countries with the highest number of published documents were England (140 documents and 3039 citations), the United States (94 documents and 2099 citations), Qatar (40 documents and 1530 citations), and Spain (90 documents and 1023 citations). In 2019, the countries with the most development were Slovakia and New Zealand. Between 2019 and 2020, the United States showed significant production, while between 2021 and 2022, there was a considerable increase in scientific output, led by England, Spain, Brazil, Italy, Canada, and Portugal. Finally, the 40 identified countries are spread across different continents, with Europe showing the greatest development, whereas Africa and Asia have lower levels of production. The different-sized nodes represent the countries, and the colors represent the interactions produced between them over time, with the darkest colors representing the countries that received the most citations in 2019–2020, and green and yellow represents those that received the most recent citations in 2022 (Figure 10).

### 3.10. Organizations

**Figure 11:**
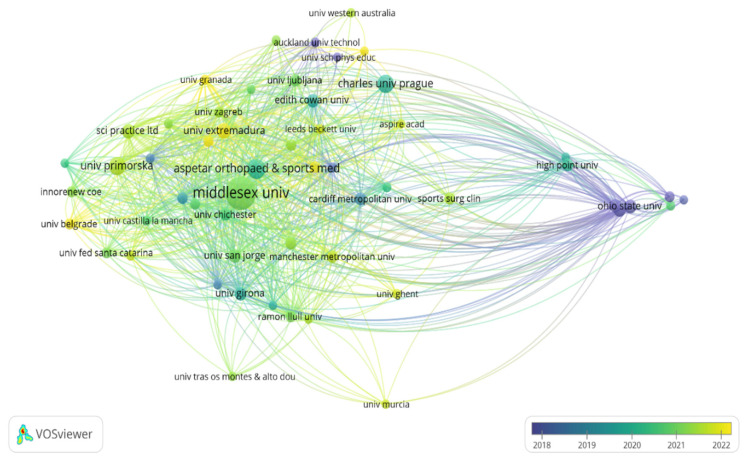
Visualization of maps and clusters of most productive organizations/institutional affiliations related to study of asymmetries in sports performance.

A total of 771 organizations were identified, 55 of which have developed at least 5 documents and received 10 citations. In terms of citations by organizations, the organizations with the highest production were “Middlesex University—United Kingdom” (72 documents and 1920 citations) and “Aspetar Orthopedic & Sport Medicine Hospital—Qatar” (25 documents and 1210 citations) (Figure 11).

## 4. Discussion

This study aimed to analyze the evolution and trends in the number of publications related to the study of asymmetries and the factors associated with sports performance. To achieve this goal, a bibliometric analysis was conducted on studies obtained from the Scopus and WoS databases. This analysis of coauthorship and keyword occurrence allows for the identification of future research directions, and provides an overview of the current state of knowledge on the selected topic. An increase of more than 100% in the number of studies published in 2018 compared with 2022–2023 is notable. The countries and institutions that focus the most on asymmetry research are England, the United States, and Qatar. Moreover, several studies in Spain, Brazil, Italy, Canada, and Portugal have been conducted recently, reflecting the growing interest in sports science regarding the effects and incidence of asymmetries in sports performance. However, more participation from countries in South America, Asia, and Africa is needed. The most common terms used in asymmetry studies are related to sports performance: performance, strength, reliability, injury, asymmetry, imbalance, muscle, knee, ACL reconstruction, return, and symmetry. The most recent trends in asymmetry research in sports performance focus on terms such as COD, sprint, speed, physical performance, and test. Recently, there has been a trend toward studying team sports, such as football and basketball, via concepts such as athlete environment interaction, acceleration, anthropometrics, patterns, and monitoring. These findings are relevant, as they indicate the incorporation of technology and other processes for monitoring sports performance.

In the analysis of the selected studies, a greater number of documents were identified on Scopus than on WoS. The evolution of the number of publications has increased in recent years, as evidenced by other bibliometric studies focusing on plyometrics [79], biomechanics [56], hamstring rehabilitation [80], and musculoskeletal injuries [81]. When the most-cited empirical studies are analyzed, the top four studies evaluate asymmetry in torque, primarily of the quadriceps [69,71,72], as an indicator of sports evaluation after returning to activity post-ACL reconstruction. These findings are in line with another bibliometric analysis that studied the most-cited articles in sports medicine and exercise, where it was concluded that most studies focus on knee-related issues, which are the most common anatomical sites when referring to ACL [82]. The increase in participation among the scientific community in studying injuries and their influence on sports performance has produced a high number of papers related to the topic; this is the main reason why production increased exponentially a few years ago.

According to Khatra et al. [82], a bibliometric study of the 100 most-cited articles revealed that research interests focused on injury prevention, rehabilitation, and biomechanics. This is similar to the results of the present study, where areas such as Orthopedics (*n* = 48; 10.19% of the total production) and Rehabilitation (*n* = 31; 6.58% of the total production) also had a significant number of articles. Asymmetry in strength has also been studied in relation to landing biomechanics to determine its effect after returning to sport [70]. These findings align with those of the study by Cone and Lee [83], which determined that torque production in the lower limbs varies with the vertical ground reaction force, generating greater asymmetry during landing than during take-off. Additionally, the most-cited review studies have focused on the impact of asymmetries on physical performance [1,51], whereas others have explored the relationship between asymmetry and injury risk [76] or exercise-induced fatigue [77]. Furthermore, some studies have focused on tests used to assess asymmetries based on strength and power production [39], and technological advancements to monitor asymmetry between limbs [78].

It has been suggested that jump height and eccentric knee torque asymmetries are associated with reduced COD performance [84]. This finding is consistent with the findings of Bishop et al. [75], who reported that asymmetries for different tests were low (≤−5.3%), with the left leg being over-represented compared with the right leg in the evaluation of jump tests. In conclusion, asymmetry studies show variations associated with the tasks performed, the metrics obtained, and individual characteristics. This aligns with the findings of the present study, which identified various concepts related to asymmetries, such as COD, sprint, speed, physical performance, and test. A review by Afonso et al. [85] concluded that bilateral asymmetries are common in sports, and that scientific evidence does not suggest that they influence sports performance or pose a significant risk of injury. On the other hand, some findings confirm that bilateral asymmetries vary with age, with younger athletes (U13, U15) being more prone to these asymmetries, although they tend to decrease at the end of adolescence [86].

Owing to multidirectional situations—such as jumps, turns, ACCs, DECs, and force–vector analysis—cooperative-oppositional sports demonstrate high force production. In this sense, bilateral symmetry is closely related not only to the specificity of the sport, but also to the inherent characteristics of performance expressed in the player’s position within such sports [87].

### 4.1. Future Perspectives and Practical Applications

In the future, there is a need for more experimental studies focused on evaluating, identifying, and recognizing asymmetries in sports performance across different sports, ages, and competitive levels, as well as analyzing them in response to their specific playing positions. Additionally, it is necessary to acknowledge that most studies have focused on elite athletes. This phenomenon would be interesting to replicate in single studies assessing both male and female athletes to address the specific needs of each context in training and competition. Additionally, evaluating asymmetries in the youth population, and evaluating how an injury can influence sports performance at the grassroots level, are recommended. Finally, the academic community is encouraged to continue developing studies focused on recognizing other performance variables related to asymmetries, to develop research proposals aimed at determining athlete profiles based on the interaction of nutritional, psychosocial, emotional, cognitive, psychological, hormonal, physical, technical, coordinative, and tactical characteristics, as well as the needs evidenced in each population context.

### 4.2. Limitations

This study has several limitations, primarily related to the heterogeneity of the population samples and the performance characteristics evaluated. Additionally, the bibliometric analysis was limited to documents indexed in the WoS and Scopus databases; thus, the language biases of these databases should be considered. Another limitation pertains to the search period, which may also limit other important findings in the study of asymmetries. In this vein, only selecting papers indexed in the JCR can limit the identification of studies to those that analyze asymmetries in sports, but exclude papers indexed in high-impact journals.

## 5. Conclusions

Two major approaches have been developed in the study of asymmetries and sports performance: one focused on analyzing differences between limbs, and the other focused on evaluating strength post surgery. These findings emphasize the importance of considering sports performance as an interdisciplinary process. Asymmetries are a determining factor in athletes’ performance development, as they influence injury risk in both the lower and upper limbs, negatively affecting motor health and performance. Therefore, proper strength training and injury rehabilitation planning should be carried out by physical trainers to prevent and improve recovery processes after injury.

Finally, the most-cited studies reported that asymmetries in drop jump height and eccentric knee torque are associated with reduced change-of-direction performance, and may influence overall sports performance. This highlights the importance of considering asymmetries as part of an interdisciplinary approach. Analyzing existing knowledge on asymmetries in sports performance could facilitate the incorporation of scientific advancements, and establish better practices for improving athletic performance and rehabilitation processes. Furthermore, there has been a systematic increase in the evaluation of asymmetries in team sports such as football and basketball. Moreover, the analysis of population samples revealed a tendency to evaluate more male athletes than female athletes, although there was also diversity in the ages of the participants. These findings invite future studies to further understand the role of asymmetries in sports performance and athlete health.

## Figures and Tables

**Table 1:** Type of document published.

Document Type	Record Count	%
Article	440	93.41
Review article	20	4.24
Meeting abstract	8	1.69
Letter	2	0.42
Retraction	1	0.21
Total	471	100

**Table 2:** Most-cited papers on review articles.

Authors and Year of Publication	Journal	Aim	Main Findings	Tot Cit	Ave per Year *
Bishop et al. [51].	Journal of Sports Sciences	Analyze the effects of interlimb asymmetries on physical and sports performance.	Jump-based asymmetries are the least conclusive tests. These strength asymmetries appear to be associated with performance in specific actions, such as jumps, sport-specific skills, and CODs.	248	41.33
Maloney [1]	Journal of Strength and Conditioning Research	Determine the relationship between asymmetry and athletic performance.	Sports asymmetries allow for describing existing bilateral differences established based on parameters associated with force production and jump height. These asymmetries do not seem to influence the assessment of athletic performance, which is due to the different methodologies used.	139	27.8
Bishop et al. [39]	Journal of Strength and Conditioning Research	Analyze the utility of strength and jumping tests that are frequently used to measure asymmetry.	Measuring asymmetries based on jump strength is a justified evaluation in sports samples, although it is necessary to consider the concept of movement variability. This is because the assessment conducted between limbs in specific actions, such as jumping and other force applications, depends on the performed task. Unilateral or bilateral jump tests can be useful for detecting asymmetries.	60	8.57
Helme et al. [76]	Physical Therapy in Sports	Synthesize the current understanding of the relationship between lower body functional asymmetry and injury risk in athletic populations.	The quality of scientific production regarding functional asymmetries of the lower limbs is moderate-to-low. Tests used to evaluate functional asymmetry as a risk factor for sports injuries are limited by the diversity of approaches and the methodological rigor of the studies. It is suggested that professionals take these observations into account when implementing sports interventions.	45	15
Heil et al. [77]	Sports Medicine—Open	Provide an overview of the current state of evidence regarding the influence of exercise-induced fatigue on interlimb asymmetries through a systematic review.	It is suggested that pre–post designs should be used to evaluate asymmetries with sport specificity and, from there, carry out an observation process at specific moments to understand physical load. The incorporation of cutting or landing movements is suggested to more realistically represent the demands of the sport. It is unclear what protocol or task selection should be employed, as the study of asymmetries depends on the context and objective of the evaluation.	33	8.25
Parkinson et al. [78]	Journal of Sports Science and Medicine	(1) Assess the appropriateness of existing indices for calculating asymmetry, (2) examine the evidence supporting the thresholds used to define asymmetry in the literature, and (3) summarize normative levels of interlimb strength asymmetry and their effects on injury and performance.	There are unequal practices when quantifying or interpreting force asymmetry between limbs using threshold limits. The normalization of commonly accepted thresholds ranging from 10 to 15% should be avoided, as they lack scientific support. Therefore, professionals should interpret asymmetries with caution.	24	8

Note. The average citations per year were calculated from the date of publication until 15 June 2024 * Tot Cit: total citations; Av per Year: average per year.

**Table 3:** Most-cited papers on empirical research articles.

Authors and Year of Publication	Journal	Aim	Main Findings	Tot Cit	Ave per Year *
Palmieri-Smith et al. [69]	American Journal of Sports Medicine	Determine whether the magnitude of quadriceps strength asymmetry influences knee and hip biomechanical symmetry, as well as functional performance and self-reported function.	ACL reconstruction negatively influences movement symmetry in the sagittal plane and functional performance, due to the quadriceps’ isokinetic strength deficit. Additionally, quadriceps strength is related to greater jump performance and knee symmetry in the sagittal plane. This highlights the importance of quadriceps symmetrical strength in different rehabilitation programs to validate the return to sports activity.	227	25.22
Schmitt et al. [70]	Medicine and Science in Sport and Exercise	Investigate the effect of quadriceps femoris (QF) strength asymmetry on knee landing biomechanics at the time of return to sport after ACL reconstruction.	Quadriceps strength deficits after anterior cruciate ligament reconstruction are associated with altered knee mechanics in bilateral landings. Individuals with quadriceps strength deficits greater than 15% in the affected limb exhibit movement asymmetries in actions such as landing, and higher loading rates on the non-injured limb.	205	22.77
Ithurburn et al. [71]	American Journal of Sports Medicine	Evaluate the influence of musculoskeletal deficiencies on movement mechanics.	Restoring quadriceps strength symmetry after an ACL injury is related to more symmetrical mechanics in single-leg drop-landing actions. This improvement enhances postoperative athletic participation and reduces the risk of a new ACL injury.	166	18.44
Zwolski et al. [72]	American Journal of Sports Medicine	Determine whether the IKDC 2000 subjective knee form score at the time of RTS is a predictor of quadriceps strength in a young, athletic population after ACLR.	The International Knee Documentation Committee (IKDC) 2000 subjective knee form is an objective assessment of quadriceps strength after anterior cruciate ligament reconstruction, making it a necessary clinical measure to determine the process of preparation and reintegration of the athlete into practice. This is because isokinetic dynamometers are not always available to objectively quantify the existing limb symmetry in quadriceps strength.	136	15.11
Bishop et al. [73]	Strength and Conditioning Research	Provide a framework for selecting the most appropriate asymmetry equation based on the selected test method, ensuring accurate calculation and interpretation.	The quantification of asymmetries between limbs can be performed using unilateral and bilateral tests. However, when selecting bilateral tests, it is necessary to choose equations that allow differences between limbs to be established independently. The BAI-1 and SI appear to be the only formulas that will accurately quantify asymmetries during bilateral tasks. For the evaluation of unilateral tests, the BSA method or percentage difference is the most suitable.	126	21
Gonzalo-Skok et al. [74]	International Journal of Sports Physiology and Performance	Compare the effects of unilateral and bilateral resistance training on single-leg power output, BLI, BLD, COD, and linear sprinting and jumping performance in young elite basketball players.	Combined unilateral training had a positive effect on reducing asymmetries between limbs, which led to an improvement in actions requiring unilateral force application compared to bilateral training. Unilateral training also showed significant changes in variables such as jumping, linear sprint, and COD, making it important to incorporate this training exercise into weekly planning.	106	15.14
Bishop et al. [75]	Journal of Strength and Conditioning Research	Determine whether interlimb asymmetries always favor the same side for common metrics across unilateral strength and jumping-based tests.	DJ asymmetries were associated with reduced COD performance in professional cricket athletes, whereas no influence was reported among soccer players. The asymmetries evaluated in the lower limbs through the SLDJ test allowed for the specification of profiles according to sport specificity. Cricket athletes showed greater jump height and RSI asymmetries, which were associated with lower COD performance in both limbs. Meanwhile, soccer players had higher values in speed, jump height, and RSI. This demonstrates the importance of evaluating asymmetries in response to the specific demands of each sport and the individuality of athletes.	105	35

Note. The average citations per year were calculated from the date of publication until 15 June 2024 * Tot Cit: total citation; Av per Year: average per year; RTS: return to sport; ACLR: anterior cruciate ligament reconstruction; IKDC: International Knee Documentation Committee; Q-LSIs: quadriceps strength limb symmetry indices; BAI-1; Bilateral Asymmetry Index 1; BSA: Bilateral Strength Asymmetry; BLI: between-limb imbalance; BLD: bilateral deficit: CODs: changes in direction; DJ: drop jump; CMJ: countermovement jump; RCOD: right change of direction; LCOD: left change of direction; MP: maximum power.; SLDJs: single-leg drop jumps; RSI: reactive strength index.

**Table 4:** Number of documents per author, considering first author.

Author	H-Index	Number of Publications	%
Bishop, C.	37	25	5.30
Malý, T.	17	8	1.69
Fort-Vanmeerhaeghe, A.	22	7	1.48
Mala, L.	14	6	1.27
Madruga-Parera, M.	13	6	1.27
Brown, S.R.	19	5	1.06
Gonzalo-Skok, O.	26	5	1.06
Total		62/471	13.16/100%

**Table 5:** Total number of documents by number of authors.

Number of Authors	Number of Publications	Percentage %
1 to 2	35	7.43
3 to 5	237	50.31
6 to 8	171	36.30
≥9	28	5.94
Total	471	100%

**Table 6:** Main characteristics of analyzed journals.

Journal	Editorial	Country	Doc	Rank	JCI Category	Quartile				JCI	Num Cit *	Ave Num Cit Pub
J Strength Cond Res	Lip Will Wil	United States	57	9/127	Sport Sciences	Q1				1.62	1230	21.57
Symmetry-Basel	MDPI	Switzerland	27	34/135	Multidisciplinary Sciences	Q2				0.86	118	4.37
Int J Environ Res Public Health	MDPI	Switzerland	23	85/325	Environmental Sciences	Q2				N/A	175	7.60
Journal of Sport Sciences	Taylor & Francis	United Kingdom	17	31/127	Sport Sciences	Q1				1.05	676	37.96
Physical Therapy in Sport		United States	15	37/127	Sport Sciences	Q2				0.97	182	12.13
American Journal of Sports Medicine	Sage Publication	United States	12	7/127	Sport Sciences	Q1				1.83	871	72.58
Int J Sports Physiol Perform	Human Kinetics	United States	12	17/121	Sport Sciences	Q1				1.27	298	24.83
Med Sci Sports Exerc	Lip Will Wil	United States	12	8/127	Sport Sciences	Q1				1.74	359	29.91
Stren Cond Res	Lip Will Wil	United States	10	50/127	Sport Sciences	Q2				0.86	338	33.80
Sports	MDPI	Switzerland	9	41/127	Sport Sciences	Q2				0.91	186	20.66
Journal of Human Kinetics	Termedia Publishing	Poland	9	50/157	Sport Sciences	Q2				0.86	143	15.88
Frontiers in Physiology	Frontiers Media SA	Switzerland	9	23/83	Physiology	Q2				1.0	49	5.44
Total: 12 Journals		Four countries	212/471	Four areas		Q1	Q2	Q3	Q4	4625		
						5	7	0	0			

Note: JCI: The Journal Citation Indicator 2023 ™ is a measure of the average Category Normalized Citation Impact (CNCI) of citable items (articles and reviews) published by a journal over a recent three-year period. It is used to help evaluate journals based on other metrics besides the Journal Impact Factor (JIF). Doc: documents; Num Cit: number of citations; Ave Num Cit Pub: average number of citations per published article; Int J Environ Res Public Health: *International Journal of Environmental Research and Public Health*; Int J Sports Physiol Perform: *International Journal of Sports Physiology and Performance*; J Strength Cond Res: *Journal of Strength and Conditioning Research*; Med Sci Sports Exerc: *Medicine and Science in Sports and Exercise*; Stren Cond Res: *Strength and Conditioning Journal*; Lip Will Wil: Lippincott Williams & Wilkins. * Times Cited WoS Core. N/A: not applicable.

**Table 7:** Total number of documents by publisher.

Editorial	Number of Documents	Total Citations
Lippincott Williams & Wilkins	87	1974
MDPI	78	558
Taylor & Francis	39	842
Sage Publication LTD	28	1007
Human Kinetics Publ Inc	22	143
Springer	22	314
Elsevier	19	133
Frontiers Media SA	17	84
Wiley	12	182
Edizion Minerva Medica	10	32
Total: 10 publishers	334/471	5269

**Table 8:** Knowledge areas.

Area *	Number of Publications	%
Sport Sciences	220	46.70
Orthopedics	48	10.19
Multidisciplinary Sciences	43	9.12
Rehabilitation	31	6.58
Physiology	25	5.30
Total: 5 areas	367/471	77.91/100%

Note. * The same article may be considered in more than one area.

## Data Availability

The data confirming the results obtained are available through the corresponding authors.
